# *Paeoniae alba* Radix Promotes Peripheral Nerve Regeneration

**DOI:** 10.1093/ecam/nep115

**Published:** 2011-01-11

**Authors:** Kun-Shan Huang, Jaung-Geng Lin, Han-Chung Lee, Fuu-Jen Tsai, Da-Tian Bau, Chih-Yang Huang, Chun-Hsu Yao, Yueh-Sheng Chen

**Affiliations:** ^1^Graduate Institute of Chinese Medical Science, China Medical University, Taiwan; ^2^Division of Neurosurgery, China Medical University Hospital, Taiwan; ^3^Department of Health and Nutrition Biotechnology, Asia University, Taiwan; ^4^Department Biomedical Imaging and Radiological Science, China Medical University, Taichung, Taiwan

## Abstract

The present study provides *in vitro* and *in vivo* evaluation of *Paeoniae alba* Radix (PR) on peripheral nerve regeneration. In the *in vitro* study, we found the PR caused a marked enhancement of the nerve growth factor-mediated neurite outgrowth from PC12 cells as well as their expression of growth associated protein 43 and synapsin I. In the *in vivo* study, silicone rubber chambers filled with the PR water extract were used to bridge a 10-mm sciatic nerve defect in rats. At the conclusion of 8 weeks, regenerated nerves in the PR groups, especially at 1.25 mg ml^−1^ had a higher rate of successful regeneration across the wide gap, relatively larger mean values of total nerve area, myelinated axon count and blood vessel number, and a significantly larger nerve conductive velocity compared to the control group (*P*  <  .05). These results suggest that the PR extract can be a potential nerve growth-promoting factor, being salutary in aiding the growth of injured peripheral nerve.

## 1. Introduction

In anastomosis, different neuro-stimulating substances have been used to investigate the possible promotion of the growth of axons in peripheral nerve to cross gaps and grow into the distal segment in a shorter period. These neuro-stimulating substances include nerve growth factor [1], brain-derived neurotrophic factor [2], collagen [3], laminin-containing gel [4], and so forth. Recently, application of combining traditional Chinese medicine and western biomedical science to nerve regeneration is another approach. For example, Liu et al. found that modified Wendan Decoction can attenuate the neurotoxicity of A*β* 25–35 and rescue neurons via suppressing apoptotic process [5]. Mohandas Rao et al. indicated that the constituents/active principles present in *Centella asiatica* fresh leaf extract have a neuronal dendritic growth stimulating property; hence, the extract can be used for enhancing neuronal dendrites in stress and neurodegenerative and memory disorders [6]. Our group has successfully demonstrated that the bilobalide, extraction of the leaves of *Ginkgo biloba*, can promote the regeneration of dissected rat sciatic nerve in a silicone rubber chamber [7]. The silicone rubber chamber filling in the bilobalide not only can aid guidance of growing nerve fibers along appropriate paths but also can enhance the precision of stump approximation. We also found that the ginsenoside Rb_1_, a main component of *Ginseng* Radix, can promote nerve growth factor-mediated nerve fiber outgrowth in rats [8]. In the present study, we tried to investigate the effect of another commonly used herbal medicine, the *Paeonia veitchii* Lynch, on peripheral nerve regeneration. *Paeoniae alba* Radix (PR), the dried root of *P. veitchii* Lynch, has long been used in Asia as an analgesic, sedative and anti-inflammation agent [9]. It is also commonly used to treat patients with cardiovascular, extravasated blood, stagnated blood and female diseases in traditional Chinese medicine [10]. Various compounds have been found in this plant, such as the monoterpene glycosides and the paeoniflorin [11]. Although the literature shows the PR has versatile functions; its neuropathic effect has seldom been characterized. Therefore, this work uses the PC12 cell line, which has been extensively adopted to study cell differentiation and neurite outgrowth [12], its neuronal characteristics upon exposure to the water extract of PR. Afterwards, we filled the extraction of PR in silicone rubber chambers for establishing a nerve bridge across a 10 mm gap for rat sciatic nerves. At the conclusion of 8 weeks, histological and electrophysiological techniques were then used to evaluate the effect of the PR on functional recovery of the regenerated nerve within the chamber.

## 2. Methods

### 2.1. Preparation of PR Extract

The PR used in this study was kindly supplied by the Chung Song Zong Pharmaceutical Co., Ltd in Kaohsiung, Taiwan. The medicinal materials were authenticated by Pharmacist Hsiao-Wu Chuang. PR (650 g) were boiled in 7.8 l distilled water at 100°C for 1 h. The aqueous solution was then filtered and concentrated using a vacuum concentrator to obtain a paste with a volume of 265 ml. Subsequently, 2.7 g of the paste was added in 45 ml of 50% MeOH. The mixture was then oscillated for 30 min by an ultrasonic oscillator. The supernatant liquid was obtained and added in 50% MeOH to a final volume of 100 ml before using. Paeoniflorin, a principle compound of PR, was purchased from Extrasynthese (France) and dissolved to a concentration of 145 *μ*g ml^−1^ in 50% MeOH.

### 2.2. Quality Control of PR

The HPLC fingerprinting method used in this study has been widely used for providing chemical information of pharmacologically active compounds in crude drugs, which is useful for the authentication and quality evaluation of the medicinal herb [13]. Chromatographic measurements were made on a Thermo Separations HPLC system comprising an ERC-31415*α* degasser, a P1000 gradient pump, an AS1000 autosampler and a UV2000 diode-array detector. Peak areas were integrated by using the software Chrom Quest 4.2.

The mobile phase was composed of H_2_O–CH_3_CN (88 : 12, v/v). A Cosmosil 5C_18_-AR-II column (250 mm × 4.6 mm i.d., 5 *μ*m, USA) was used. The flow-rate was 1.0 ml min^−1^ with UV absorbance detection at 230 nm. The operation was carried out at room temperature. The retention times of the biomarker substance was 18.6 min for paeoniflorin. The linearity of the peak area (*y*) versus concentration (*μ*g ml^−1^) curve for paeoniflorin was used to calculate the contents of the biomarker substances in the PR.

### 2.3. Cell Culture

PC12 cells were maintained at 37°C in a humidified atmosphere of 5% CO_2_ and 95% air in Ham's F12K medium supplemented with 15% horse serum, 2.5% fetal calf serum, 2 mM HEPES and 2 mM _L_-glutamine. Since the PC12 cells adhered quite poorly to the plastic, they were cultured on collagen-coated tissue culture dishes. PC12 cells were then induced to undergo neuronal differentiation by treatment with 50 ng ml^−1^ of NGF (#N0513, Sigma, St Louis, MO, USA) in the culture medium with 1.25, 12.5, 125 *μ*g ml^−1^ of the PR extract. Cells that had been exposed to the vehicle alone (culture medium and NGF only) were the control.

Morphometric analysis was conducted on digitized images of live cells obtained under phase-contrast illumination using an Olympus HAL100 inverted microscope that was connected to a Nikon digital camera. Images of 35 fields were obtained per well. The number of differentiated cells was determined by visually examining the field and counting cells that had at least one neurite, whose length was 3 times of their cell body diameter. The count was expressed as a proportion of the total number of cells in the field. Neurite growth was monitored by manually tracing the length of the longest neurite (using Image-Pro Lite Version 3.0 software) for all cells in the field that had an identifiable neurite, and whose complete neurite arbor could be visualized. A minimum of 1500 cells were studied for each data point.

### 2.4. Western Blot Analysis

Growth associated protein 43 (GAP-43) and synapsin I were quantified using western blot analysis. Cells were cultured in 75 cm^2^ tissue culture flasks and treated collagen-coated for 6-well plates (1 × 10^5^ cells/well). PC12 cells were plated at a high density to obtain sufficient amount of protein for analysis. The cells were treated with the NGF and different concentrations of the PR extract on Days 0 and 2. The cells were then collected on Day 3 by gentle shaking of the flask, washed twice and sonicated for 15 s in ice-cold lysate buffer (62.5 mM Tris, pH 6.8, 2% sodium dodecyl sulfate (SDS), 50 mM 1,4-dithio-_D,L_-threitol). The cell lysate was centrifuged at 10 000 g for 10 min at 4°C and the resulting supernatant was saved for protein analysis and western blot. The protein content of cells was determined by the Bradford protein assay using the protein-day kit (Bio-Rad, Hercules, CA). A commercially available bovine serum albumin (Sigma Chemical, St Louis, MO, USA) was used as a standard. Changes in optical density were monitored at 595 nm. For western blotting, the supernatant was added to an equal volume of Laemmli sample buffer (62.5 mM Tris, pH 6.8, 60% glycerol, 2% SDS and 0.6% bromophenol blue) and heated to 95°C for 5 min. Proteins (10 g total protein per lane) were separated by SDS-PAGE on 10% polyacrylamide gels and were transferred onto polyvinylidene difluoride membranes. The membranes were then incubated with a polyclonal rabbit GAP-43 and synapsin I antibody, respectively (Santa Cruz Biotechnology, Inc., Santa Cruz, CA; 1 : 1000) overnight at 4°C. Immunoreactivity was detected using peroxidase-linked secondary antibody and enhanced chemiluminescence detection reagent (Pierce, Rockford, IL). Images were collected and band density was analyzed using a Fluor-S MultiImager and Quantity One software (Bio-Rad). Experiments were repeated four to six times using cultures made on Day 3. Data were expressed as a percent of control level of protein within an individual experiment. Densitometric analysis of immunoblots was performed using an AlphaImager 2200 digital imaging system (Digital Imaging System, San Leandro, CA, USA).

### 2.5. Surgical Preparation of Animals

Twenty-eight adult Sprague-Dawley rats underwent placement of silicone chambers. The animals were anesthetized with an inhalational anesthetic technique (AErrane, Baxter, USA). Following skin incision, fascia and muscle groups were separated by blunt dissection, and the right sciatic nerve was severed into proximal and distal segments. The proximal stump was then secured with a single 9-0 nylon suture through the epineurium and the outer wall of the silicone rubber chamber (1.47 mm ID, 1.96 mm OD; Helix Medical, Inc., Carpinteria, CA). The distal stump was then secured in the other end of the chamber. Both the proximal and distal stumps were secured to a depth of 1 mm into the chamber, leaving a 10 mm gap between the stumps.

Animals were divided into four groups. In the first group (*n* = 7), the chambers were filled with normal saline as controls. Chambers in groups 2 (*n* = 7), 3 (*n* = 7) and 4 (*n* = 7) were filled with the PR extract at concentrations of 1.25, 12.5 and 125 mg ml^−1^, respectively. The concentrations of PR extract used *in vivo* were 1000 times of that used *in vitro*. The volume of the chamber lumen was about 25.5 *μ*l. These fillings were injected through a micropipette into the lumens by passing the tip of the needle into the silicone rubber chambers. Loading was performed as slowly as possible to prevent the formation of air bubbles. After the loading, the muscle layer was re-approximated using 4-0 chromic gut sutures, and the skin was closed with 2-0 silk sutures. All animals were housed in temperature (22°C) and humidity (45%) controlled rooms with 12 h light cycles. They had access to food and water *ad libitum*. All chambers remained in place for 8 weeks, during which the nerves were re-exposed and the chambers studied to determine the presence of regenerated nerve across the 10-mm gap. All animals were maintained in facilities approved by the China Medical University for Accreditation of Laboratory Animal Care, according to the regulations and standards of the National Science Council of Health of the Republic of China.

### 2.6. Electrophysiological Methods

Following the implantation period of 8 weeks, all animals were re-anaesthetised and the sciatic nerve exposed. The sciatic nerve was stimulated with supramaximal stimulus intensity through a pair of needle electrodes placed directly on the sciatic nerve trunk, 5-mm proximal to the transection site. Amplitude, latency and area of the evoked muscle action potentials (MAPs) were recorded from gastrocnemius muscles with micro-needle electrodes linked to a computer system (Biopac Systems, Inc., USA). The latency was measured from stimulus to the takeoff of the first negative deflection. The amplitude and the area under the MAP curve from the baseline to the maximal negative peak were calculated. The MAP was used to calculate the nerve conductive velocity (NCV), which was carried out by placing the recording electrodes in the gastrocnemius muscles and stimulating the sciatic nerve proximally and distally to the silicone rubber conduit. The NCV was then calculated by dividing the distance between the stimulating sites by the difference in latency time.

### 2.7. Histological Methods

Sciatic nerve sections were extracted from middle of the regenerated nerve in the chamber. Following fixation, the nerve tissue was post-fixed in 0.5% osmium tetroxide, dehydrated and embedded in spurs. The tissue was then cut to a thickness of 5 *μ*m using a microtome with a dry glass knife, stained with toluidine blue. All tissue samples were observed under a light microscope (Olympus IX70, Olympus Optical Co., Ltd, Japan). An image analyzer system (Image-Pro Lite, Media Cybernetics, USA), coupled to the microscope then counted the blood vessels and calculated the cross-sectional area of each the nerve section at magnifications of between 40x and 400x. At least 30–50% of the nerve section area was randomly selected from each nerve specimen at a magnification of 400x to count the axons. The axon counts were extrapolated by using the area algorithm to estimate the total number of axons in each nerve. All data are expressed as mean  ±  standard deviation. Statistical comparisons between groups were made using the one-way ANOVA. A *P*-value of <.05 was considered statistically significant.

## 3. Results

The quality of the PR was monitored by the amount of paeoniflorin in the water extract. According to the results of the HPLC analysis, the PR contained about 64.1 mg g^−1^ of the paeoniflorin.

### 3.1. Relationship of Morphology and Protein Expression

**Figure 1:**
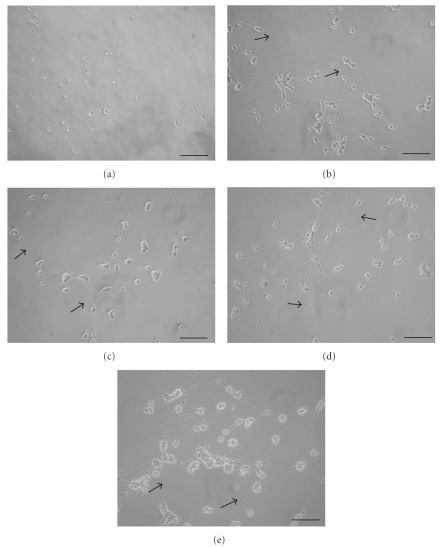
Effects of PR extract on the morphology of PC12 cells. The cells were treated for 72 h without PR extract in the absence of NGF (a), without PR extract in the presence of NGF (b), with PR extract in the presence of NGF ((c) PR at 1.25 *μ*g ml^−1^; (d) PR at 12.5 *μ*g ml^−1^; (e) PR at 125 *μ*g ml^−1^). It was noted that the PR could stimulate the NGF-induced neurite outgrowths (arrows) from PC12 cells. Scale bars = 100 *μ*m.

**Figure 2:**
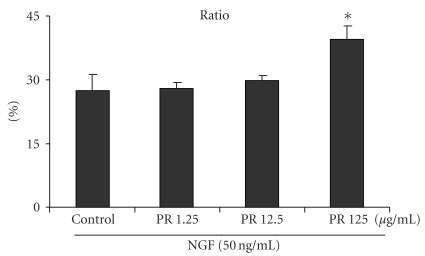
Effects of PR on the NGF-induced neurite outgrowth. Neurite-bearing cells with more than three cell diameters are expressed as a ratio against the total cultured PC12 cells. *P < .05 versus treatment with vehicle alone.

**Figure 3:**
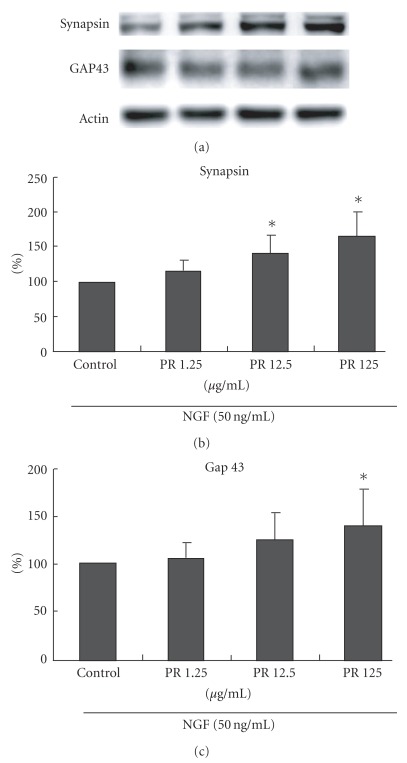
Representative immunoblots for synapsin I and GAP-43 and quantification of both the protein levels in NGF-treated PC12 cells with different concentrations of PR extract relative to the level of the controls. *P < .05 versus treatment with vehicle alone.

Under the microscope, almost no PC12 cells exhibited neurite outgrowths of larger than one cell diameter when cultured in the absence of NGF (Figure 1(a)). In comparison, NGF stimulated a few neurite outgrowths from PC12 cells (Figure 1(b)). Additionally, PC12 cells exposed to PR with NGF all formed long neurites, which extended to the neighboring cells of distances even more than three cell diameters (Figures 1(c)–1(e)). These results are supported by quantitative assays that reveal the PR significantly potentiated NGF-induced neurite outgrowth from PC12 cells, especially those in the group with 125 *μ*g ml^−1^ of PR at *P* < .05 (Figure 2). In the western blot analysis, the group treated with PR could dramatically increase the expression of GAP-43 and synapsin I in NGF-treated PC12 cells (Figure 3). The enhanced percentage of PC12 cells showed dose dependence after co-treatment of PR at 1.25–125 *μ*g ml^−1^ with 50 ng ml^−1^ of NGF.

### 3.2. Percentage of Nerve Cable Formation in Bridging Chambers

Gross examination of the silicone rubber chambers at 8 weeks revealed successful regeneration in groups 2–4 with PR, in 86% (6 of 7), 86% (6 of 7) and 43% (3 of 7) of the animals, respectively, which exhibited a regenerated nerve cable across the 10-mm gap. In comparison, 57% (4 of 7) of the animals in the controls exhibited such regenerated nerve cables.

### 3.3. Evaluation of Maturity of Regenerated Nerves

**Figure 4:**
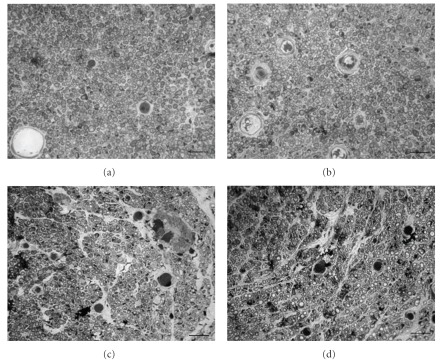
Light micrographs of regenerated nerve cross-sections from group 2 ((a) PR at 1.25 mg ml^−1^), group 3 ((b) PR at 12.5 mg ml^−1^), group 4 ((c) PR at 125 mg ml^−1^) and control group ((d) saline). Scale bars = 30 *μ*m.

**Figure 5:**
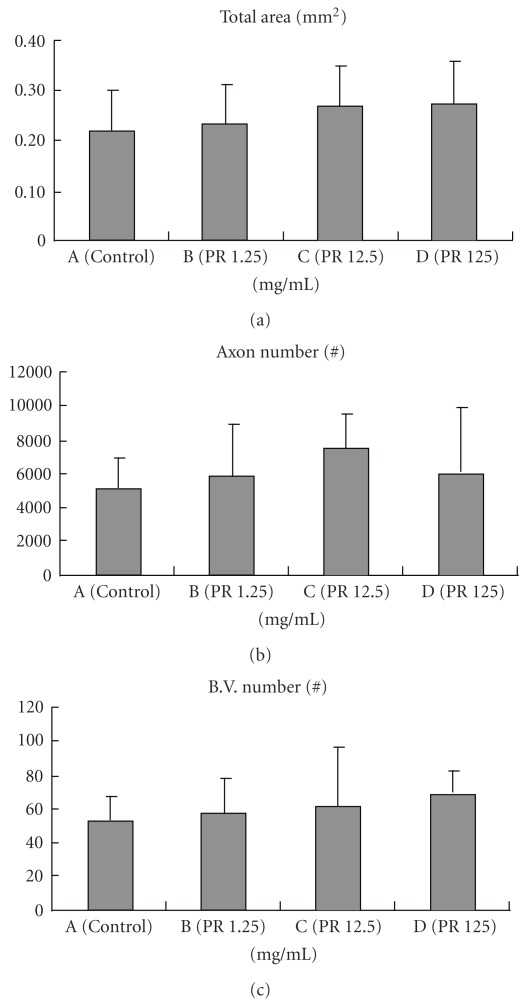
Morphometric analysis from the regenerated nerves in the silicone rubber chambers, including total nerve area (a), myelinated axon count (b) and blood vessel number (c).

Figures 4(a)–4(c) presents representative cross sections of regenerated nerve specimens. Regenerated nerves selected from all the PR groups exhibited a similar ultrastructural organization. The epineurial and perineurial regions of the regenerated nerves comprised primarily a collagenous connective tissue matrix in which circumferential cells that resembled perineurial cells and fibroblasts were observed. Myelinated axons were numerous in the endoneurium and easily defined by the toluidine blue staining surrounding the myelin sheath. Nuclei of Schwann cells were interspersed among these axons. Additionally, blood vessels were numerous in the epineurium and in the endoneurial areas of the nerve. The regenerates in the control group had a similar structure (Figure 4(d)); however, morphometric measurements showed the mean values of total nerve area, myelinated axon count and blood vessel number were all larger in the three PR groups than in the controls (Figures 5(a)–5(c)).

### 3.4. Electrophysiological Examination of Regenerated Nerves

**Figure 6:**
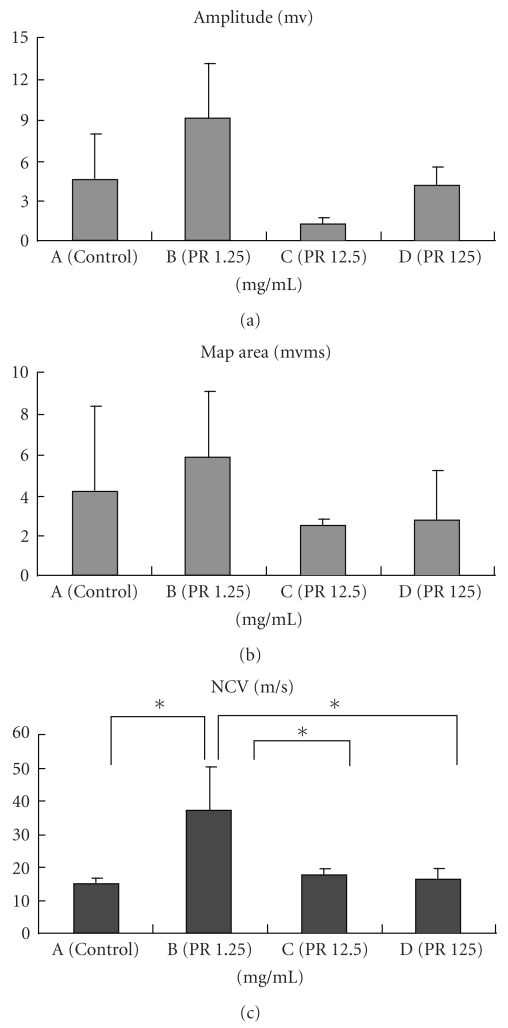
Analysis of the evoked MAPs, including peak amplitude (a) area under the MAP curves (b) and NCV (c). *P < .05 when PR group at 1.25 mg ml^−1^ compared with the other three groups.

With respect to electrophysiology (Figures 6(a)−6(c)), excitability and conductivity were obvious in the nerve cables with regenerated axons. The regenerated nerve cables treated with 1.25 mg ml^−1^ of PR had a significantly larger NCV as compared with those in the other three groups (*P* < .05).

## 4. Discussion

Peripheral nerve regeneration involves a series of highly specialized healing processes [14]. Therefore, a wide variety of methods are adopted in anastomosis studies, according to the goals of the work. The use of artificial tubes to bridge a severed nerve enables regenerative processes to be studied under controlled experimental conditions.

As seen in this study, the PR water extract had nerve-differentiating effects *in vitro*, considerably promoting the differentiation of neurite-bearing cells and the expression of GAP-43 in cultures supplemented with NGF. The GAP-43 is a marker for growth cones [15]. Western blot analyses of cell lysates demonstrated that the NGF-induced increase in GAP-43 was further enhanced with PR treatment, confirming that PR had promoting effect on new sprout formation. In addition, it was found that the PR could increase the expression of synapsins, which are abundant phosphoproteins essential for regulating neurotransmitter release [16, 17].

To clearly reveal the influence of PR extract on nerve regenerative process *in vivo*, silicone rubber was used as the guide material in the present study. Silicone rubber is one of the most acceptable materials used to make the bridging chambers because of its stable property. It has been demonstrated that silicone rubber tubes are well tolerated in humans even after 3 years of implantation [18]. In addition, since silicone rubber is non-permeable, it can provide an isolated environment for the regenerating fibers. Therefore, the only biochemical factors that may influence regeneration are the cells, fluids and the PR extract within the chamber.

As a result, we found that administration of an appropriate dosage of PR could significantly enhance the formation of a nerve cable across the wide nerve gap in the silicone rubber chamber. Eighty-six percent of the animals in 1.25 and 12.5 mg ml^−1^ of PR-treated groups had cables that grew across the gap. In comparison, only 57% of the animals in the control group exhibited such bridging cables. However, the high dose PR (125 mg ml^−1^) completely reversed this positive effect of growth-promoting capability and inhibited nerve regeneration. Only 43% of the animals treated with the high dose PR had regenerated cables within the silicone rubber chambers. These results imply whether a proper dosage of PR used plays a critical factor in deciding, if it can sustain nerve regeneration over long gaps. Excessively loaded PR in the tube could provoke adverse responses to the recovery of regenerated nerves.

In the morphometric data, we found that the PR could enhance nerve growth within the nerve cable with relatively larger mean values of total nerve area, myelinated axon count and blood vessel number as compared to the controls. We speculate a possible mechanism for this promoted regeneration is that the PR may exert some protective functions on regenerating neurons. In the literature, it has been found that the paeoniflorin, a characteristic main principal bioactive component of PR could activate adenosine A_1_ receptors [9]. The adenosine A_1_ receptors are highly expressed on macrophages and neurons [19], and the activation of the adenosine A_1_ receptors could attenuate neuro-inflammation and demyelination in the mice model of multiple sclerosis [20]. Similarly, the paeoniflorin has been found that it could ameliorate cerebral hypo-perfusion-related learning dysfunction and prevent neuron damage [21]. We believe the protective properties of the paeoniflorin underlie the potential beneficial effects of the PR on regenerating nerves.

In addition to the morphometric differences, we found the application of PR, especially at 1.25 mg ml^−1^ could significantly increase the NCV as compared with the controls. Since nerve fibers are components of the peripheral nervous system, we believe the relatively larger nerve areas and more myelinated axons in the PR-treated nerves are the reason causing the acceleration of their NCV. But, we also found large variations in other electrophysiological measurements, such as the amplitude and the area under the MAP curve. These may result from serious gastrocnemius muscle atrophy even though the muscle fibers had been reinnervated during 8 weeks of recovery. In addition, misdirected regeneration, that is, a sensory fascicle could be anastomosed to a motor one or vice versa, could result in the inconsistent electrophysiological evaluation [22].

## 5. Conclusion

**Figure 7:**
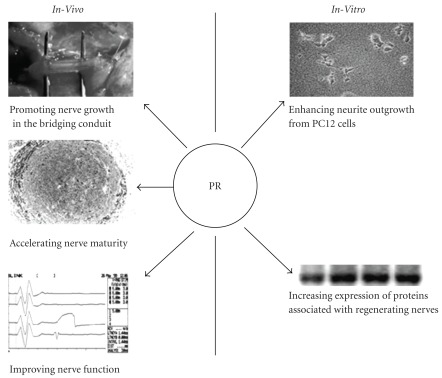
Summary of the effects of PR on PC12 cells and regenerating peripheral nerves.

In conclusion, the current work reports the first use of the PR extract could significantly promote NGF-induced neurite outgrowth with increasing expression of GAP-43 and synapsin I from PC12 cells. In addition, PR extract filled in silicone rubber chamber do have a significant increased effect on the nerve repair and regeneration during the initial 8th week time point. Thus, the PR extract can be a nerve growth-promoting factor which can be applied for peripheral nerve regeneration (Figure 7).

## Funding

China Medical University (contract no CMU96-062); the National Science Council of the Republic of China, Taiwan (contract no NSC96-2628-E-039-011-MY3).
